# Serum levels of interleukin-17 and adiponectin are associated with infrapatellar fat pad volume and signal intensity alteration in patients with knee osteoarthritis

**DOI:** 10.1186/s13075-016-1088-9

**Published:** 2016-08-26

**Authors:** Kang Wang, Jianhua Xu, Jingyu Cai, Shuang Zheng, Weiyu Han, Benny Antony, Changhai Ding

**Affiliations:** 1 Department of Rheumatology and Immunology, Arthritis Research Institute, the First Affiliated Hospital of Anhui Medical University, 218 Jixi Street, Hefei, China; 2 Menzies Institute for Medical Research, University of Tasmania, Private Bag 23, Hobart, Tasmania 7000 Australia

**Keywords:** Osteoarthritis, Infrapatellar fat pad, Adipokines, Cytokines, Cartilage, Epidemiology

## Abstract

**Background:**

In the present study, we sought to generate hypotheses regarding the associations of serum levels of interleukin (IL)-17, adiponectin, and resistin with magnetic resonance imaging-measured infrapatellar fat pad (IPFP) size and signal intensity alterations in patients with knee osteoarthritis (OA).

**Methods:**

A total of 170 subjects with symptomatic knee OA (mean age 55.4 years, range 34–74, 88.2 % females) were included. IPFP volume was measured on T1-weighted spoiled gradient-recalled acquisition in the steady state images and was computed by using a software program. IPFP high signal intensity (grades 0–3) was assessed on T2-weighted fast spin echo images. Serum IL-17, adiponectin, and resistin levels were measured using an enzyme-linked immunosorbent assay.

**Results:**

In multivariable analyses, serum IL-17 was negatively associated with IPFP volume (β = −0.185, 95 % CI −0.337 to −0.034) but positively associated with the severity of IPFP signal intensity alteration (OR 1.23, 95 % CI 1.06–1.42) after adjustment for age, sex, weight, and height. Serum adiponectin was positively associated with IPFP volume (β = 0.016, 95 % CI 0.001–0.032) but negatively associated with IPFP signal intensity alteration (OR 0.99, 95 % CI 0.98–1.00) after adjustment for covariates. Resistin was positively associated with IPFP signal intensity alteration (OR 1.13, 95 % CI 1.04–1.23) but not with IPFP volume. The significant associations of adiponectin or resistin disappeared after further adjustment for IL-17; in contrast, the significant associations of IL-17 remained after further adjustment for adiponectin.

**Conclusions:**

While serum IL-17 and resistin were associated with reduced IPFP volume and/or increased abnormal signal intensity alteration, serum adiponectin had opposite associations that were largely through IL-17. These findings suggest that serum adipocytokines may have a role to play in IPFP changes of knee OA.

## Background

Osteoarthritis (OA) is a common joint disease with multiple pathogenetic mechanisms, affecting the whole joint. Obesity is considered one of the potent risk factors for developing OA [1]. Although the pathogenesis of OA is not well known, recent studies indicate that obesity-related proinflammatory and metabolic factors contribute to OA progression [2]. Infrapatellar fat pad (IPFP), a local adipose tissue in knee joints with an abundance of adipocytes, immune cells, vessels, and nerve fibers, may be an active joint tissue involved in the initiation and progression of knee OA [3].

IPFP, also known as Hoffa’s fat pad, is an intracapsular and extrasynovial structure [4]. It is thought to have primarily a biomechanical function of absorbing forces generated in the knee [5], and thus to have a protective effect physiologically. Indeed, in two recent studies, researchers reported that larger IPFP size was associated with reduced knee abnormal structural changes and symptoms [5, 6]. On the contrary, IPFP signal intensity alteration assessed using magnetic resonance imaging (MRI) was positively associated with the prevalence and/or incidence of knee pain, cartilage defects, bone marrow lesions, and radiographic osteoarthritis (ROA) in older adults [7], and thus it may have a detrimental effect. IPFP has been considered an important source of cytokines and adipokines (i.e., adipocytokines) in OA [8]. IPFP tissues acquired from patients with knee OA can produce cytokines such as interleukin (IL)-1β, tumor necrosis factor (TNF)-α, IL-6, IL-8, IL-17, basic fibroblast growth factor, and vascular endothelial growth factor (VEGF) [8–10]. It can also produce various adipokines such as leptin, resistin, and adiponectin [11]. Researchers in an *in vitro* study reported that conditioned media from cultured white adipose tissue from OA IPFP that contained leptin could induce cartilage collagen release and increase matrix metalloproteinase (MMP)-1 and MMP-13 expression in chondrocytes, and thus had catabolic effects on cartilage [12].

Although the local inflammatory profile of IPFP tissue has been investigated *in vitro*, the associations between serum adipocytokines and IPFP changes have not been examined in epidemiological or clinical studies. Osteoarthritic IPFP explants can release high protein levels of IL-17, resistin, and adiponectin [10]. While serum IL-17 and resistin (proinflammatory adipocytokines) concentrations were significantly higher in patients with knee OA than in control subjects [13], serum levels of adiponectin (an anti-inflammatory adipocytokine) were associated with decreased disease severity of knee OA [14]. It is unknown if IPFP measures such as size and signal intensity alteration are associated with increased or decreased release of systemic IL-17, resistin, and adiponectin in knee OA. We expected that IPFP size would be associated with decreased levels of serum IL-17 and resistin and an increased level of serum adiponectin, but IPFP high signal intensity would be associated with increased levels of serum IL-17 and resistin and a decreased level of serum adiponectin in patients with knee OA. The aim of this study was therefore to generate hypotheses regarding the associations between serum levels of IL-17, adiponectin, and resistin and the volume and signal intensity alteration of IPFP measured using MRI in patients with symptomatic knee OA.

## Methods

### Study design and patients

We consecutively enrolled into the Anhui Osteoarthritis Study 205 patients with OA who fulfilled the American College of Rheumatology criteria for the classification of clinical knee OA at the Outpatient Clinics, Department of Rheumatology, First Affiliated Hospital of Anhui Medical University, from January 2012 to November 2013. We excluded institutionalized persons, patients with rheumatoid arthritis or other inflammatory diseases, patients with severe knee OA who were planning to have knee arthroplasty in 2 years (this study was ongoing with 2 years of follow-up), and patients with contraindications to MRI (including metal sutures, presence of shrapnel, iron filings in the eye, and claustrophobia). The study was approved by the First Affiliated Hospital of Anhui Medical University Ethics Committee, and all participants signed the informed consent forms.

### Anthropometrics

Weight was measured to the nearest 0.1 kg (with shoes, socks, and bulky clothing removed) using a single pair of electronic scales (RGZ-120; Jiangsu Province, China) that were calibrated using a known weight at the beginning of each clinic. Height was measured to the nearest 0.1 cm by using a stadiometer with shoes, socks, and headgear removed. Body mass index (BMI) (weight [kg]/height [m^2^]) was calculated.

### Radiographic OA assessment

All patients underwent knee radiography. The 15-degree flexion, standing, anteroposterior view image was taken in the symptomatic knees (the severer one if both knees were affected; the right one if both knees were equally painful). Radiographic assessment was performed by a radiology specialist using the Kellgren-Lawrence (K-L) grading system (grades 0–4): grade 0, normal; grade 1, no joint space narrowing (JSN), suspicious osteophytes; grade 2, suspicious JSN, mild osteophytes; grade 3, definite JSN, moderate osteophytes, and/or subchondral bone sclerosis; and grade 4, marked JSN, large osteophytes, and/or severe subchondral bone sclerosis [15]. Radiographic OA was defined as a K-L grade ≥2.

### Assessments of IPFP volume and signal intensity changes

MRI of the selected knee was performed with a 3.0-T whole-body magnetic resonance imaging unit (Signa HDxT 3.0T; GE Healthcare, Little Chalfont, UK), using a commercial transmit/receive extremity coil. The following sequence and parameters were used:

1. A T1-weighted fat saturation three-dimensional spoiled gradient-recalled acquisition in the steady state (SPGR), flip angle 30 degrees; repetition time 31 milliseconds; echo time 6.71 milliseconds; field of view 16 cm; 60 partitions; 512 × 512-pixel matrix; acquisition time 11 minutes, 56 milliseconds; and 1 acquisition (Sagittal images were obtained at a partition thickness of 1.5 mm and an in-plane resolution of 0.31 × 0.31 [512 × 512 pixels].)
2. A T2-weighted fat saturation two-dimensional fast spin echo, flip angle 90 degrees, repetition time 3067 milliseconds, echo time 112 milliseconds, field of view 16 cm, 15 partitions, 256 × 256-pixel matrix (Sagittal images were obtained at a slice thickness of 4 mm with an interslice gap of 1.0 mm. Images were checked for image noise and structural abnormalities interfering with segmentation.)

IPFP area was measured by manually drawing disarticulation contours around the IPFP boundaries on T1-weighted SPGR MRI scans using OsiriX software (Pixmeo Sàrl, Bernex, Switzerland). IPFP volume was computed by using the software program. IPFP signal intensity alteration on T2-weighted MRI studies was recorded if hyperintense signal alterations were observed within the IPFP. Signal intensity alteration, defined as discrete areas of increased signal within the IPFP, was graded as follows: grade 0, none; grade 1, <10 % of the region; grade 2, 10–20 % of the region; and grade 3, >20 % of the region [16]. All images were grouped together and read in randomized order by two readers, with the reader blinded to subjects’ information. Intraobserver reliability was measured in 30 subjects, with an intraclass correlation coefficient of 0.95 (95 % CI 0.89–0.97). Interobserver reliability was 0.94 (95 % CI 0.85–0.97).

### Serum adipocytokine measurements

Morning fasting blood samples were collected from patients. Serum was separated, aliquoted into plastic storage tubes, and stored at −80 °C until analysis. Serum levels of IL-17, adiponectin, and resistin were measured by enzyme-linked immunosorbent assay (ELISA) (eBioscience, San Diego, CA, USA) kits according to the manufacturer’s instructions. The optical density was measured at 450 nm using an automatic ELISA reader (Sunrise; Tecan, Männedorf, Switzerland). The limits of detection for IL-17, adiponectin, and resistin were 0.5 pg/ml, 0.01 ng/ml, and 3.1 pg/ml, respectively, and the coefficients of variation were 7.1 %, 4.2 %, and 5.1 %, respectively.

### Data analysis

Student’s *t* test, the *χ*^2^ test, and the Mann-Whitney *U* test was used to compare means, proportions, and medians, respectively. Pearson’s correlations or Spearman’s analyses were used to analyze the correlations of serum levels of IL-17 (log-transformed) with serum levels of adiponectin and resistin (log-transformed) or K-L grading. Linear regression analyses were used to examine the associations between IL-17, adiponectin, or resistin and IPFP volume (the dependent variable that was normally distributed) before and after adjustment for covariates including age, sex, weight, and height. Ordinal regression analyses were used to examine the associations between IL-17, adiponectin, or resistin and IPFP signal intensity alteration (the dependent variable). Standard diagnostic checks of model fit and residuals were routinely done, and data points with large residuals and/or high influence were investigated for data errors. A *p* value <0.05 (two-tailed) or a 95 % CI not including the null point was regarded as statistically significant. All statistical analyses were performed using SPSS 13.0 for Windows (SPSS, Chicago, IL, USA).

## Results

A total of 170 subjects (88.2 % females) aged between 34 and 74 years (mean 55.4 years) were included in the analyses, and another 35 subjects were excluded from the study because of incomplete data. There were no significant differences in demographic factors between those included and excluded (data not shown). The median IL-17 level was 2.00 pg/ml, the mean IPFP volume was 20.46 ml (range 12.39–42.90 ml), and the IPFP signal intensity alteration mean value was 0.78 (range 0–3). There were 73 % patients who had established ROA.

Characteristics of the subjects based on the median value of IL-17 are presented in Table 1. Patients with higher and lower levels of IL-17 were similar in terms of sex, BMI, height, weight, knee OA, IPFP signal intensity alteration, IPFP volume, and resistin level. However, patients with higher levels of IL-17 were older and had lower adiponectin levels. Serum levels of IL-17 were significantly associated with serum levels of adiponectin (*r* = −0.234, *p* = 0.002) and resistin (*r* = 0.165, *p* = 0.032). Serum IL-17 and resistin were not associated with K-L grade, but adiponectin was negatively correlated with K-L grade (ρ = −0.211, *p* = 0.006).

**Table 1:** Characteristics of participants (split by median level of interleukin 17)

	Total (*n* = 170)	IL-17 ≤ median (*n* = 85)	IL-17 > median (*n* = 85)	*p* Values
Age, years^a^	55.45 (8.25)	54.07 (8.17)	56.66 (8.39)	**0.044**
Female sex, %^b^	88.2	89	87	0.634
Height, cm^a^	159.00 (6.89)	159.09 (7.01)	158.58 (6.88)	0.638
Weight, kg^a^	64.83 (10.56)	64.23 (9.62)	65.09 (10.70)	0.591
BMI, kg/m^2a ^	25.64 (3.85)	25.35 (3.20)	25.87 (3.88)	0.341
K-L grade, %^b^				0.268
1	27.9	25.7	28.6	
2	41.9	50.0	35.1	
3	25.7	20.3	31.2	
4	4.5	4.1	5.2	
Knee ROA, %^b^	72.1	74.3	71.4	0.689
IPFP signal intensity alteration, %^b^	43.1	44.2	40	0.298
IPFP volume, mm^3a^	20.46 (5.01)	20.57 (5.53)	20.42 (4.88)	0.855
Adiponectin, pg/ml^c^	18.67 (3.94–53.07)	35.08 (11.36–71.26)	12.82 (3.00–46.94)	**0.001**
Resistin, ng/ml^c^	2.37 (1.40–5.57)	2.18 (1.45–4.86)	2.81 (1.32–5.78)	0.532

*Abbreviations: BMI* body mass index, *K-L* Kellgren-Lawrence, *IL* interleukin, *ROA* radiographic osteoarthritis, *IPFP* infrapatellar fat pad

ROA was defined as a K-L grade ≥2. IL-17 median level 2.00 pg/ml (interquartile range 1.56–2.82). Data in bold denote statistically significant results

^a^ *t* tests were used for mean (SD)

^b^χ^2^ tests were used for the proportions

^c^Mann-Whitney *U* tests were used for median (interquartile range)

**Fig. 1:**
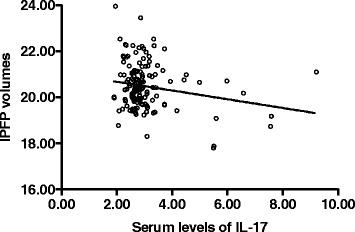
Association between serum IL-17 and infrapatellar fat pad volume in patients with knee osteoarthritis. *r* = −0.199, *p* = 0.015 after adjustment for age, sex, height and weight. *IL* interleukin, *IPFP* infrapatellar fat pad

Serum level of IL-17 was significantly and negatively associated with IPFP volume after adjustment for age, sex, weight, and height (Table 2, Fig. 1), and this association remained unchanged after further adjustment for adiponectin (Table 2). Serum level of adiponectin was significantly and positively associated with IPFP volume after adjustment for age, sex, weight, and height, but this became nonsignificant after further adjustment for IL-17 (Table 2). There was no significant association between resistin and IPFP volume before or after adjustment for potential confounders.

**Table 2:** Association between adipocytokines and infrapatellar fat pad volume

Adipocytokines	Multivariable^a^ β (95 % CI)	*p* Value	Multivariable β (95 % CI)	*p* Value
IL-17	**−0.19 (−0.34 to −0.03)**	**0.017**	**−0.16 (−0.32 to −0.01)** ^b^	**0.047**
Adiponectin	**0.02 (0.01 to 0.03)**	**0.039**	0.01 (−0.01 to 0.03)^c^	0.190
Resistin	−0.07 (−0.20 to 0.06)	0.303	0.02 (−0.01 to 0.18)^c^	0.773

*IL* interleukin

Dependent variable: infrapatellar fat pad volume. Independent variable: cytokines. Data in bold denote statistically significant results

^a^Adjusted for age, sex, weight and height

^b^Further adjustment for adiponectin

^c^Further adjustment for IL-17

**Fig. 2:**
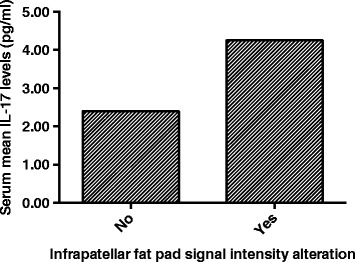
Association between serum IL-17 and infrapatellar fat pad signal intensity alteration in patients with knee osteoarthritis. Serum IL-17 levels were higher in those with signal intensity alteration (*p* = 0.001)

Serum levels of IL-17 were significantly and positively associated with severity of IPFP signal intensity alteration before and after adjustment for age, sex, weight, and height, and this association remained significant after further adjustment for adiponectin (Fig. 2, Table 3). Adiponectin had a negatively significant and resistin a positively significant association with IPFP signal intensity alteration before and after adjustment for age, sex, weight, and height, but these associations became nonsignificant after further adjustment for IL-17 (Table 3).

**Table 3:** Association between adipocytokines and infrapatellar fat pad signal intensity alteration

Adipocytokines	Multivariable^a^ OR (95 % CI)	*p* Value	Multivariable OR (95 % CI)	*p* Value
IL-17	**1.23 (1.06–1.42)**	**0.007**	**1.20 (1.03–1.38)** ^b^	**0.017**
Adiponectin	**0.99 (0.98–1.00)**	**0.042**	0.99 (0.98–1.00)^c^	0.177
Resistin	**1.13 (1.04–1.23)**	**0.004**	1.11 (1.00–1.23)^c^	0.054

*IL* interleukin

Dependent variables: IPFP signal intensity alteration (0–3). Independent variable: adipocytokines. Data in bold denote statistically significant results

^a^Adjusted for age, sex, weight, and height

^b^Further adjustment for adiponectin

^c^Further adjustment for IL-17

The significant associations of IL-17 and resistin remained significant, but associations of adiponectin became nonsignificant, after adjustment for K-L grade (data not shown).

## Discussion

To our knowledge, this is the first epidemiological study to illustrate the relationship between serum levels of adipocytokines and IPFP volume and signal intensity alteration in patients with knee OA. After adjustment for age, sex, weight, and height, we found that higher serum IL-17 level was associated with lower IPFP volume and increased IPFP signal intensity alteration (indicating poorer IPFP quality). Higher adiponectin level was associated with higher IPFP volume and better IPFP quality, but higher serum resistin level was associated only with poorer IPFP quality. These suggest that serum levels of adipocytokines are associated with IPFP pathophysiology in knee OA.

The role of IPFP in OA progression is inconclusive. In our previous studies, we have reported that IPFP size may have a protective role in knee OA structural changes: Greater IPFP maximal area is associated with greater cartilage volume and lesser cartilage defects, bone marrow lesions, and R OA cross-sectionally and/or longitudinally in older adults [5, 6]. Although researchers in one study reported that IPFP volume was correlated only with age in patients with knee OA and might not be related to the progression of OA [17], we recently reported that, in patients with knee OA, IPFP volume was associated with greater cartilage volume and fewer cartilage defects, bone marrow lesions, and osteophytes [18]. The signal intensity changes in IPFP were positively associated with the prevalence and/or incidence of knee pain, cartilage defects, bone marrow lesions, and R OA in older adults [7], suggesting that abnormal quality of IPFP is detrimental for knee symptoms and structures. This is supported by a recent study demonstrating that synovitis measured using IPFP signal intensity change (Hoffa’s synovitis) is associated with the development of radiographic knee OA [19]. The severity of inflammation in IPFP measured by dynamic contrast-enhanced MRI was associated with the severity of pain in knee OA [20].

Animal or *in vitro* studies have indicated a potentially important role of IPFP in mediating knee intra-articular inflammation. IPFP produced an elevated level of inflammatory cytokines, growth factors, and adipokines in a high-fat diet-induced murine OA model, and the expression levels of the adipokines were significantly correlated with expression of TNF-α, VEGF, and transforming growth factor β [21]. Rates of IL-6 expression and secretion were higher in IPFP tissue than in subcutaneous adipose tissue, indicating that the IPFP cytokine profile could play a role in paracrine inflammation via the local production of IL-6 and contribute to damage in adjacent cartilage in patients with OA [8]. IPFP explants from patients with OA could produce a variety of cytokines, and the production was increased by local cytokine stimulation [9].

Preliminary evidence has shown that adipocytokines such as IL-17 may have roles to play in knee OA. A significant proportion (up to 20 %) of chondrocytes in cartilage samples from patients with OA expressed IL-17R [22]. An in vitro study suggested that IL-17 induced the release of chemokines by chondrocytes and synovial fibroblasts, contributing to cartilage breakdown and synovial infiltration in OA [23]. Researchers in a cross-sectional study reported that serum IL-17 concentrations were significantly higher in patients with knee OA than in control subjects, and synovial IL-17 concentrations were positively correlated with K-L grade and Lequesne index in patients with knee OA [24]. So far, there have been no studies illustrating the relationship between serum IL-17 levels and IPFP changes in patients with knee OA. We found that serum level of IL-17 was negatively associated with IPFP volume and positively with IPFP high signal intensity in patients with knee OA. According to the biopsies from the infrapatellar region in knee OA, chronic low-grade inflammation was detected as signal changes on fluid-sensitive MRI sequences [25]. Our results suggest that serum IL-17 is associated with IPFP pathophysiology, but it is unknown if IL-17 contributes to IPFP inflammation or if abnormal IPFP may produce IL-17, which could play a role in OA pathogenesis. The causal relationship needs to be confirmed in future longitudinal studies.

The role of adiponectin in OA is largely unclear. In vitro findings suggested that adiponectin increased nitric oxide and MMP production in human OA chondrocytes mainly via the AMP-activated protein kinase/c-Jun N-terminal kinase pathway, which would lead to accelerated degradation of OA cartilage matrix ex vivo [26]. Adiponectin can also induce IL-6 secretion in a cultured chondrogenic cell line [27]. In contrast, adiponectin levels in both plasma and synovial fluid decreased significantly as the severity of OA (evaluated by K-L grading) increased in humans [28]. Serum adiponectin levels were negatively associated with radiographic progression in patients with hand OA [29, 30]. Furthermore, adiponectin expression in IPFP was higher than in subcutaneous adipose tissue in patients with knee OA [8] and was higher in patients with OA in end stage than in early stage [31]. In our present study, the serum levels of adiponectin were positively associated with IPFP volume and negatively with IPFP signal intensity alteration, indicating that adiponectin may have a role in IPFP of knee OA. This association was dependent on IL-17 and K-L grade, suggesting that this may be mediated largely by IL-17 or ROA.

The biological effects of resistin in OA remain controversial. Recently, researchers in one study reported that resistin could upregulate the expression of multiple chemokines, cytokines, and matrix-degrading genes that are involved in OA pathobiology [32]. Researchers in a cross-sectional study reported that serum resistin levels in patients with hand ROA were higher than in patients with nonradiographic hand OA and control subjects, and were associated with radiographic bone erosion in hand OA [33]. In the present study, we did not find a significant association between resistin and IPFP volume, but we found a significantly positive association between resistin and IPFP signal intensity alteration, suggesting a potential role of resistin in abnormal IPFP changes of knee OA.

The present study has some limitations. First, it was a cross-sectional study designed to generate hypotheses, and the causal relationship cannot be interpreted. Second, the subjects were recruited from the clinics consecutively rather than from the community randomly, so the results may not generalizable to patients with general knee OA. Third, the signal intensity alteration of IPFP was assessed by unenhanced MRI, which was nonspecific, and its pathology is largely unclear [34]. Last, the levels of adipocytokines were measured in serum rather than in synovial fluid, so their local effects are unknown.

## Conclusions

While serum IL-17 and resistin were associated with reduced IPFP volume and/or increased abnormal signal intensity alteration, serum adiponectin had opposite associations, which were largely through IL-17. These results suggest that serum levels of adipocytokines may have a role to play in IPFP changes of knee OA.

## Abbreviations

BMI: Body Mass Index
IL: Interleukin
ELISA: Enzyme-linked Immunosorbent Assay
IPFP: Infrapatellar Fat Pad
JSN: Joint Space Narrowing
K-L: Kellgren-Lawrence
MMP: Matrix Metalloproteinase
MRI: Magnetic Resonance Imaging
OA: Osteoarthritis
ROA: Radiographic Osteoarthritis
SPGR: Spoiled Gradient-recalled Acquisition in the Steady State
TNF: Tumor Necrosis Factor
VEGF: Vascular Endothelial Growth Factor
